# Plyometric jump training effects on the physical fitness of individual-sport athletes: a systematic review with meta-analysis

**DOI:** 10.7717/peerj.11004

**Published:** 2021-03-01

**Authors:** Silvia Sole, Rodrigo Ramírez-Campillo, David C. Andrade, Javier Sanchez-Sanchez

**Affiliations:** 1 Faculty of Nursery and Physiotherapy, University of Lleida, Lleida, Spain; 2 GRECS Research Group, IrB Lleida, Lleida, Spain; 3 Department of Physical Activity Sciences, Universidad de Los Lagos, Santiago, Chile; 4 Centro de Investigación en Fisiología del Ejercicio, Facultad de Ciencias, Universidad Mayor, Santiago, Chile; 5 Centro de Fisiología y Medicina de Altura, Facultad de Ciencias de la Salud, Universidad de Antofagasta, Antofagasta, Chile; 6 Research Group Planning and Assessment of Training and Athletic Performance, Pontifical University of Salamanca, Salamanca, Spain

**Keywords:** Human physical conditioning, Resistance training, Exercise therapy, Physical education and training, Muscles, Plyometric exercise, Sports, Martial arts, Stretch reflex, Athletic performance

## Abstract

**Background:**

The aim of this study is to conduct a systematic review with meta-analysis to explore the effects of plyometric jump training (PJT) on the physical fitness of individual sport athletes (ISA).

**Methods:**

Following the Preferred Reporting Items for Systematic Reviews and Meta-Analyses guidelines, we searched through PubMed, Web of Science, and SCOPUS electronic databases. We included controlled studies that incorporated a PJT intervention among ISA (with no restriction for age or sex), that included a pre-to-post intervention assessment of physical fitness (e.g., sprint; jump). From the included studies, relevant data (e.g., PJT and participants characteristics) was extracted. We assessed the methodological quality of the included studies using the PEDro scale. Using a random-effects model, meta-analyses for a given outcome was conducted. Means and standard deviations for a measure of pre-post-intervention physical fitness from the PJT and control groups were converted to Hedges’ g effect size (ES). Heterogeneity was assessed using the *I*^2^ statistic. The risk of bias was explored using the extended Egger’s test. The statistical significance threshold was set at *p* < 0.05. Moderator analyses were conducted according to the sex, age and sport background of the athletes.

**Results:**

Twenty-six studies of moderate-high methodological quality were included (total participants, *n* = 667). Compared to controls, PJT improved vertical jump (ES = 0.49; *p* < 0.001; *I* = 0.0%), linear sprint (ES = 0.23; *p* = 0.032; *I*^2^ = 10.9%), maximal strength (ES = 0.50; *p* < 0.001; *I^2^* = 0.0%) and endurance performance (ES = 0.30; *p* = 0.028; *I^2^* = 11.1%). No significant effect was noted for sprint with change of direction (ES = 0.34; *p* = 0.205; *I^2^* = 70.9%). Athlete’s sex, age and sport background had no modulator role on the effect of PJT on vertical jump, linear sprint, maximal strength and endurance performance. Among the included studies, none reported adverse effects related to the PJT intervention.

**Conclusions:**

PJT induces small improvements on ISA physical fitness, including jumping, sprinting speed, strength and endurance.

## Introduction

Physical fitness is of critical importance to several sports, particularly in individual sports. For individual sport athletes (ISA), adequate levels of explosive strength, maximal strength, sprinting, acceleration, deceleration, change of direction, and endurance, are key factors potentially affecting athlete’s performance (Aughey et al., 2013; Coyle, 1995; Kovacs, 2006). Several training methods are routinely used by ISA in order to boost physical fitness, such as plyometric jump training (PJT) (Bazyler et al., 2015; Ebben, Hintz & Simenz, 2005). PJT is characterized by jumping drills with different ground contact times, usually involving a slow muscle-tendon stretch-shortening cycle (SSC) or a fast SSC (Ramirez-Campillo et al., 2020c). Jumping drills involving slow SSC are performed with ground contact times >250 ms, large lower-limb joint excursion, and low stiffness (e.g., counter drop jump) (Bobbert, 1990; Bobbert, Huijing & Van Ingen Schenau, 1987; Komi, 2003). On the other hand, jumping drills involving fast SSC are performed with ground contact times <250 ms, short lower-limb joint excursion, and high stiffness (e.g., bounce drop jump) (Bobbert, 1990; Bobbert, Huijing & Van Ingen Schenau, 1987; Komi, 2003). During both slow and fast SCC, the accumulation of elastic energy facilitates greater mechanical work (i.e., explosive strength) production in subsequent actions (Komi & Gollhofer, 1997; Radnor et al., 2017; Taube, Leukel & Gollhofer, 2012). Indeed, the SSC of the muscle-tendon complex optimize the rate of force development, the relative force per-motor unit recruited, and therefore, muscle power (Radnor et al., 2017). In this sense, PJT, through enhancement of the SSC and associated neuro-mechanical mechanisms (Markovic & Mikulic, 2010), may facilitate improvements in physical fitness.

There is compelling evidence showing that PJT may improve physical fitness regardless of age and sex (Andrade et al., 2018; De Villarreal et al., 2009; De Villarreal, Requena & Cronin, 2012; De Villarreal, Requena & Newton, 2010). However, the studies that explored the effects of PJT on the physical fitness in ISA have reported inconsistent results, with benefits in some (Bishop et al., 2009; Ramirez-Campillo et al., 2014), but not all studies (Blagrove et al., 2018; Bogdanis et al., 2019; Ramirez-Campillo et al., 2020c). Furthermore, one additional methodological limitation of most PJT intervention studies is their relatively small sample size (Ramirez-Campillo et al., 2018; 2020c). A 2020 PJT scoping review including 420 articles reported that an average of ~10 participants was included per study (Ramirez-Campillo et al., 2020c). In PJT studies conducted among ISA, the average sample size was ~13 participants per study, suggesting that some studies may be statistically underpowered to find significant effects. Moreover, even if significant effects emerge from studies with a small sample size, their replicability would be limited (Abt et al., 2020). This problem of underpowered studies may partially be resolved by conducting a meta-analysis. To date, several systematic reviews and meta-analysis that assess the effects of PJT on different components of physical fitness have been published. These analyses have provided evidence that PJT is effective for inducing large improvements in vertical jump ability (ES = 0.84) (De Villarreal et al., 2009), strength (ES = 0.97) (De Villarreal, Requena & Newton, 2010), and sprint capacity with (ES = 0.96) (Asadi et al., 2016) or without change of direction (COD; ES = 0.97) (De Villarreal, Requena & Cronin, 2012). To date, however, no previous meta-analysis has systematically explored the effects of PJT on the physical fitness of ISA. Moreover, previous meta-analyses incorporated participants across a range of different sports. Because the effects of PJT may vary depending on the sports background of the athlete, findings from these studies cannot be generalised to ISA (Asadi et al., 2016; De Villarreal, Requena & Cronin, 2012; Taylor et al., 2018; Vlachopoulos et al., 2018).

Therefore, considering: (i) the increased scientific awareness of the relevance of PJT, evidenced by a 25-fold increase in PJT-related scientific publications from 2000 to 2017 (Ramirez-Campillo et al., 2018), and (ii) the apparent inconsistent findings of isolated studies on the effects of PJT interventions on the physical fitness of ISA, we aimed to conduct a systematic review with meta-analysis to explore the effects of PJT on the physical fitness of ISA.

## Methods

This meta-analysis was conducted following the guidelines of the Preferred Reporting Items for Systematic Reviews and Meta-Analyses (Liberati et al., 2009).

### Search strategy

For this meta-analysis, we searched through PubMed/MEDLINE, Web of Science (Core Collection), and SCOPUS electronic databases from the inception of indexing until 30 April 2020. Potentially relevant keywords were collected through experts’ opinions, literature reviews and organized vocabulary (i.e., Medical Subject Headings: MeSH). In the PubMed/MEDLINE database, the following search syntax was used: randomized controlled trial (Publication Type) AND training (Title/Abstract) OR plyometric (Title/Abstract) OR plyometric exercise (MeSH Terms) AND sports (MeSH Terms). We excluded studies based on the review of the title, abstract, or when needed, the full-text. Following the main systematic searches, lead author’s personal libraries were also examined. Grey literature sources in the form of conference proceedings were also considered provided that the full-text was available. The search process was conducted by RRC and SS. Any disagreement was resolved with the intervention of a third author (DA).

### Eligibility criteria

We included studies that satisfied the following inclusion criteria: (i) incorporated a PJT intervention, defined as lower-body unilateral or bilateral bounds, jumps, and hops that commonly utilize a pre-stretch or countermovement that initiates the usage of the SSC (Chu & Myer, 2013; Moran, Ramirez-Campillo & Granacher, 2018; Ramirez-Campillo et al., 2018); (ii) included cohorts of ISA, with no restriction for age or sex; (iii) PJT was compared with a control group of athletes that did similar training, with the only difference between the groups being the PJT intervention; and (iv) the study included a pre-to-post intervention assessment of physical fitness (e.g., sprint; jump) (Girard, Mendez-Villanueva & Bishop, 2011).

### Data extraction

Data was extracted using a pre-defined form created in Microsoft Excel (Microsoft Corporation, Redmond, WA, USA), including: the first author’s last name and the year of study publication, PJT treatment description, description of the control comparison, type of randomization, and the total sample size. We also extracted data regarding the participants’ sex, age (years), body mass (kg), height (m) and previous experience with PJT. If applicable, information about the type and level (e.g., professional, amateur) of sport practice was also retrieved. Regarding PJT characteristics, extracted data included training frequency (days/week) and training duration (weeks), intensity level and marker of intensity, jump box height (cm), total number of jumps completed during the PJT intervention, types of jump drills performed, combination (if applicable) of PJT with another form of training type, rest time between sets, repetitions, and between sessions, type of jumping surface, type of progressive PJT overload (e.g., volume-based; technique-based), training period of the year (e.g., in-season), portion of the regular training replaced (if applicable) with PJT (e.g., PJT replaced 20% of the technical-training load), tapering strategy before post-intervention assessment (if applicable).

### Methodological quality assessment

We assessed the methodological quality of the included studies using the Physiotherapy Evidence Database (PEDro) scale. This scale evaluates different aspects of the study design, such as participant eligibility criteria, randomization, blinding, attrition, and reporting of data. There are 11 items on the PEDro checklist, but item 1 is not included in the total score. Therefore, the maximum possible score on the checklist was 10. Based on the summary score and in line with previous meta-analyses that focused on PJT (Ramirez-Campillo et al., 2020d; Stojanović et al., 2017), studies that scored ≤3 points were considered as being of “poor quality”, studies scoring 4 or 5 points were considered as being of “moderate quality” and studies that scored 6–10 points were considered as being of “high quality”.

### Statistical analysis

Although two studies can be used in meta-analyses (Valentine, Pigott & Rothstein, 2010), considering reduced sample sizes are common in the sport science literature (Pigott, 2012), including in PJT studies (Abt et al., 2020; Lohse et al., 2020; Ramirez-Campillo et al., 2018, 2020c), meta-analysis was only conducted when >3 studies were available (Garcia-Hermoso, Ramirez-Campillo & Izquierdo, 2019; Moran, Ramirez-Campillo & Granacher, 2018; Skrede et al., 2019). Means and standard deviations (SD) for a measure of pre-post-intervention physical fitness from the PJT and control groups were converted to Hedges’ g effect size (ES). The data were standardized using post score SD. In all analyses, we used the random-effects model to account for differences between studies that might impact the treatment effect (Deeks, Higgins & Altman, 2008; Kontopantelis, Springate & Reeves, 2013). The ES values are presented alongside their respective 95% confidence intervals (CIs). Calculated ES were interpreted using the following scale: <0.2, trivial; 0.2–0.6, small; >0.6–1.2, moderate; >1.2–2.0, large; >2.0–4.0, very large; >4.0, extremely large (Hopkins et al., 2009). For studies that included more than one intervention group, the sample size in the control group was proportionately divided to facilitate comparison across the multiple groups (Higgins, Deeks & Altman, 2008). Heterogeneity was assessed using the *I*^2^ statistic. *I*^2^ values of <25%, 25–75% and >75%, were considered to represent low, moderate and high levels of heterogeneity, respectively. The risk of bias was explored using the extended Egger’s test (Egger et al., 1997). In case of a significant Egger’s test the trim and fill method from Duval and Tweedie was applied for adjustment (Duval & Tweedie, 2000). All analyses were carried out using the Comprehensive Meta-Analysis program (version 2; Biostat, Englewood, NJ, USA). The statistical significance threshold was set at *p* < 0.05.

### Moderator analysis

Based on previously described methods (Sánchez et al., 2020), we used a random-effects model and independent computed single factor analysis, in order to identify potential sources of heterogeneity likely to influence the effects of PJT, including athletes sex, age and sport background. For age-related analyses, participants were divided in adult (i.e., ≥18 years of age) compared to youth (<18 years of age). For sex-related comparisons, participants were categorized as female, male or combined male-female groups. For sport background-related comparisons, athletes were grouped in a specific sport discipline (e.g., runners; gymnasts) when ≥3 studies provided data for the same sport, otherwise the athletes from different sport backgrounds were mixed for analyses.

In addition, moderator analyses were conducted for linear sprint and maximal strength according to type of test. Specifically, moderator analysis was conducted according to linear sprint test of short duration (i.e., <20 s) compared to moderate duration (i.e., >20 s). Regarding maximal strength tests, moderator analysis was conducted according to dynamic compared to isometric tests.

## Results

### Study selection

**Figure 1:**
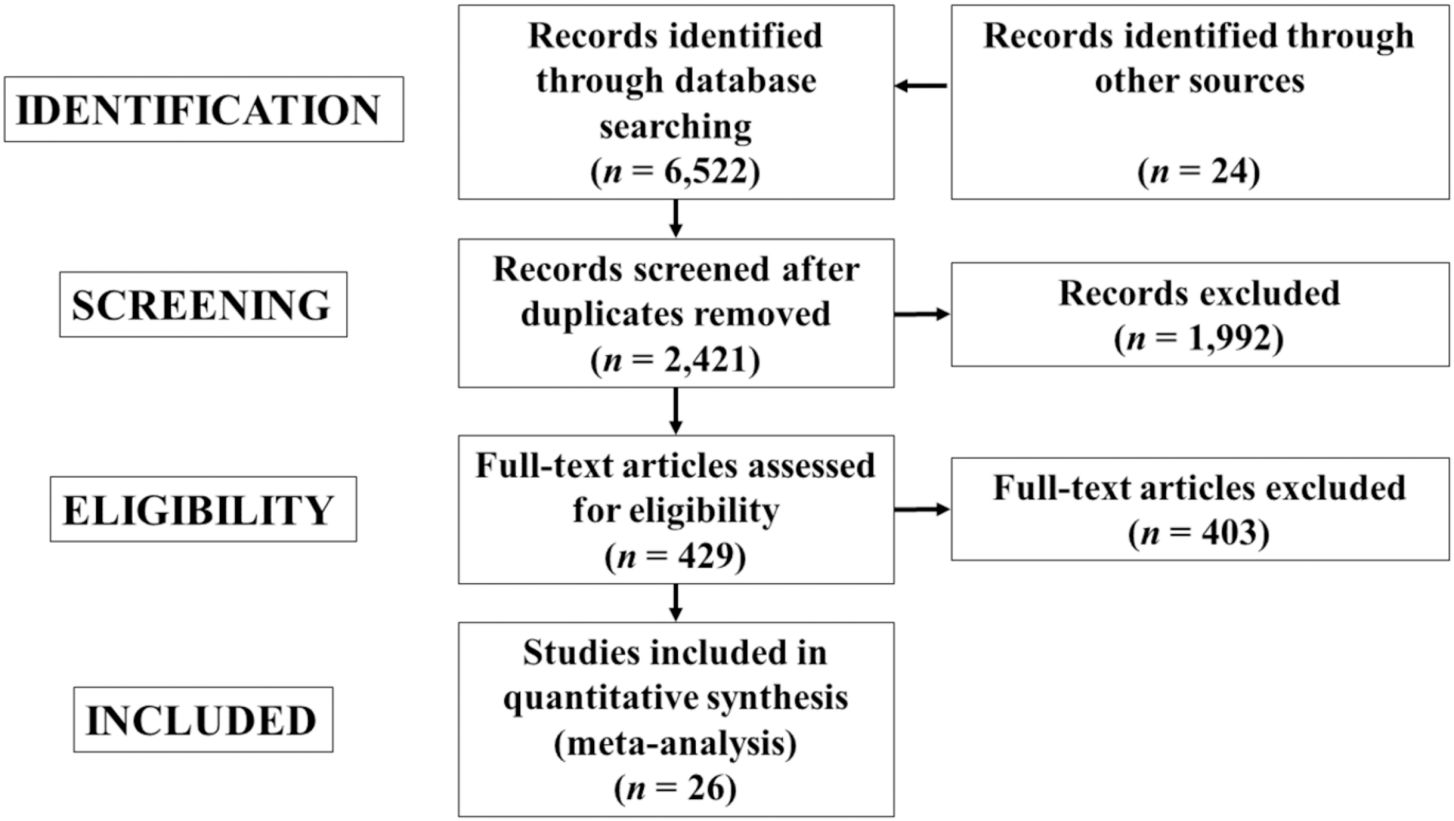
Flow diagram of the search process.

From records that were initially identified (*n* = 6,546), and after excluding the duplicates and studies based on title, abstract, or full-text, 26 studies were included in the meta-analysis (Table 1). Figure 1 provides a diagram of the study selection process. The included studies involved 27 individual experimental groups and 345 participants and 322 participants in the 27 control groups. The characteristics of the participants from the studies are displayed in Table 1, while the programming parameters of the PJT interventions are presented in Table 2. The physical fitness outcomes for both the control and PJT groups are presented in Table 3.

**Table 1:** Characteristics of included study participants in experimental groups.

Authors, year	Ran	*n*	Gender	Age*	BM (kg)*	Height (m)	SPT	Sport practiced	Fitness	TP	Replace	Tapering
Ache-Dias et al. (2016)	Yes	9	F/M	24.3	63.1	160	No	Runners	Normal	NA	No	Yes
Agostini et al. (2017)	Yes	15	F	15.4	NR	169	NR	Gymnastics	High	All season	No	NR
Alvarez et al. (2012)	Yes	5	M	24.2	68.1	172	Yes	Golfers	High	IS	Yes	No
Behringer et al. (2013)	Yes	10	M	15.5	65.2	177	No	Tennis	Normal-moderate	NR	No	No
Berryman, Maurel & Bosquet (2010)	Yes	11	M	29	74.6	178	No	Runners	Moderate-high	NR	No	Yes
Bishop et al. (2009)	Yes	11	M	13.1	50.6	163	NR	Swimmers	Moderate-high	PS	No	No
Blagrove et al. (2018)	Yes	9	F/M	16.5	57.8	170	No	Runners	Normal-moderate	OS	No	No
Bogdanis et al. (2019)	Yes	33	F	8.1	28.7	129	No	Gymnasts	Moderate	PS	Yes	No
Chelly, Hermassi & Shephard (2015)	Yes	14	M	11.7	43.0	158	No	Runners (up to 3-km)	Moderate	IS	No	No
Cossor, Blanksby & Elliot (1999)	NR	19	F/M	11.7	47.4	159	NR	Swimmers	Normal-moderate	IS	NR	No
Fernandez-Fernandez et al. (2016)	No	30	M	12.5	44.2	157	No	Tennis	Moderate	IS	Yes	NR
Giovanelli et al. (2017)	Yes	13	M	36.3	71.9	176	No	Runners	Moderate-high	NR	No	NR
Hall, Bishop & Gee (2016)	Yes	12	F	12.5	40.5	146	NR	Gymnasts	Moderate	NR	No	No
Li et al. (2019)	NR	10	M	22.2	63.1	178	NR	Runners	High	NR	Yes	No
Ludstrom, Betker & Ingraham (2017)	Yes	11	F/M	20.8	73.5	NR	No	Runners	Moderate-normal	IS	Yes	Yes
Park Lee & Lee (2014)	Yes	5	M	17.6	77.2	174	NR	Throwers	NR	NR	No	No
Pellegrino, Ruby & Dumke (2016)	Yes	11	F/M	32.5	68.2	172	No	Runners	Normal	NA	No	No
Potdevin et al. (2011)	Yes	12	F/M	14.3	50.0	161	NR	Swimmers	Moderate	PS	No	No
Ramirez-Campillo et al. (2014)	Yes	17	F/M	22.1	60.0	NR	No	Runners	High	IS	No	No
Redondo et al. (2014)	Yes	6	M	24.8	70.4	173	Yes	Fencers	High	IS	Yes	No
Salonikidis & Zafeiridis (2008)	Yes	16	M	21.1	71.7	174	Yes	Tennis	Normal-moderate	NR	NR	NR
Saunders et al. (2006)	Yes	7	M	23.4	67.6	NR	No	Runners	High	NR	No	No
Sedano et al. (2013)	Yes	6	M	24.1	68.5	181	No	Runners	High	PS	Yes	No
Turner, Owings & Schwane (2003)	Yes	10	F/M	31.0	65.4	170	No	Runners	Normal	NA	No	No
Vlachopoulos et al. (2018)	Yes	19	M	14.5	57.2	170	NR	Swimmers	Normal-high	NR	NR	Yes
		14		14.1	57.7	168		Cyclists				
Wilson et al. (1993)	Yes	13	NR	22.1	71.6	174	NR	Recreationally RT	Normal	NA	No	No

**Notes:**

* Mean values for groups.

Abbreviations descriptions ordered alphabetically. Fitness was classified as in the recent review by Ramirez-Campillo et al. (2020d); BM, body mass; F, female; IS, in-season; M, male; NA, not applicable; NR, not reported; OS, off-season; PS, pre-season; Ran, randomised; SPT, indicates if the participants had previous systematic experience with plyometric jump training; TP, training period.

**Table 2:** Characteristics of plyometric jump training (PJT) programs.

Authors, year	Freq	Dur	Int	BH (cm)	NTJ	Tply	Combined	RBS (s)	RBR (s)	RBTS (h)	Tsurf	PO
Ache-Dias et al. (2016)	2	4	Max	NA	1,200 s	CMJ + VJ (continuous 30 s)	No	300	NA	48	NR	V
Agostini et al. (2017)	2–3	48	Max	NR	NR	Mix	No	NR	NR	NR	NR	NR
Alvarez et al. (2012)	2	18	NR	NA	720	Hurdles	RT + SS	240	NR	48–120	NR	No
Behringer et al. (2013)	2	8	NR	NA	NR	Mix	No	20–60	0–1	55–78	NR	Comb
Berryman, Maurel & Bosquet (2010)	1	8	Max	20–60	240	DJ	No	180	NR	168	NR	V
Bishop et al. (2009)	2	8	NR	43–64	1,768	Mix	No	60–90	NR	NR	NR	Comb
Blagrove et al. (2018)	2	10	NR	NR	868 + 540 m	Mix	RT + sprint	90	NR	48–96	NR	Comb
Bogdanis et al. (2019)	2	8	NR	20–30	2,464	Mix	No	30 – 300	NR	48-120	Gymnastics carpet	Comb
Chelly, Hermassi & Shephard (2015)	3	10	Max	30–40	1,800	DJ + hurdles	No	NR	5	48	NR	Comb
Cossor, Blanksby & Elliot (1999)	3	20	NR	NR	18,000–27,000	NR	No	NR	NR	48–72	Land	T
Fernandez-Fernandez et al. (2016)	2	8	Max	NR	1,199	Mix	No	15–90	NR	NR	NR	Comb
Giovanelli et al. (2017)	3	12	NR	NA	NR	Mix	RT + core	0–30	NR	≥48	Stable + unstable	NR
Hall, Bishop & Gee (2016)	2	6	NR	15–30	417	Mix	No	60	NR	NR	Concrete	Comb
Li et al. (2019)	3	8	NR	40–60	1,296	Mix	No	240–480	NR	48-72	NR	No
Ludstrom, Betker & Ingraham (2017)	1	12	Max	NR	1,075	Mix	Sprints	≥60	NR/NA	168	NR	Comb
Park Lee & Lee (2014)	3	12	NR	NA	25,200	Mix	No	NR	NR	NR	NR	V
Pellegrino, Ruby & Dumke (2016)	2–3	6	Max	NR	900–3,420	Mix	No	NR	NR	NR	NR	Comb
Potdevin et al. (2011)	2	6	NR	40	2,146	Mix	No	NR	NR	NR	NR	Comb
Ramirez-Campillo et al. (2014)	2	6	Max	20–60	720	DJ	No	120	15	≥48	Wood	No
Redondo et al. (2014)	2	6	Max	NA	432	VJ	RT	180	NR	≥48	NR	No
Salonikidis & Zafeiridis (2008)	3	9	NR	20–40	NR	Mix (unilateral)	No	180–240	60–120	NR	NR	NR
Saunders et al. (2006)	2–3	9	Max	NR	852 + 2,700 m	Mix	RT	NR	NR	NR	Mix	Comb
Sedano et al. (2013)	2	12	NR	NA	2,880	Mix	RT	25–300	NR	≥48	Hard synthetic	NR
Turner, Owings & Schwane (2003)	3	6	Max	NA	1,287	Mix	No	NR	NR	NR	Inclined (6–8%) + flat	V
Vlachopoulos et al. (2018)	3–4	36	NR	NA	8,880	CMJ	No	NA	NA	NR	Hard Surface	Comb
Wilson et al. (1993)	2	10	Max	20–80	288–480	DJ	No	180	NR	NR	NR	Comb

**Note:**

Abbreviations descriptions ordered alphabetically. BH, box height; CMJ, countermovement jump; DJ, drop jump; Dur, duration (weeks); Freq, training frequency (days/week); Int, intensity; Max, maximal, involving either maximal effort to achieve maximal height, distance, reactive strength index, velocity (time contact or fast stretch-shortening cycle), or another marker of intensity; Mix, mixed PJT involved a combination of two or more of the following jumping drills: vertical, horizontal, bilateral, unilateral, repeated, non-repeated, lateral, cyclic, sport-specific (SS), slow stretch-shortening cycle, fast stretch-shortening cycle; NA, non-applicable; NR, non-clearly reported; NTJ, number of total jumps (usually counted as jumps per each leg); PO, progressive overload, in the form of either volume (i.e., V), intensity (i.e., I), type of drill (i.e., T), or a combination of these (Comb); PS, pre-season; PJT, plyometric jump training; RBR, rest time between repetitions (only when the PJT programme incorporated non-repeated jumps); RBS, rest time between sets; RBTS, rest between training sessions; Repl, replace, denoting if the athletes replace some common drills from their regular training with PJT drills. If not, the PJT load was added to their regular training load; RT, resistance training; Surf, type of surface used during the intervention; TP, training period; Tply, type of PJT drills used; Tsurf, type of surface; VJ, vertical jump.

**Table 3:** Study groups and their physical fitness.

Author (year)	Test	PJT, before^α^			Control, before			PJT, after			Control, after		
		Mean	SD	*n*	Mean	SD	*n*	Mean	SD	*n*	Mean	SD	*n*
Ache-Dias et al. (2016)	Jump (CMJ, cm)	37.74	5.24	9	40.02	6.52	9	39.52	5.06	9	39.37	6.65	9
	Strength (KE, concentric; N.m)	173.77	39.73	9	180.03	66.28	9	178.97	37.92	9	175.44	66.88	9
	Endurance (vVO2peak, k.h^−1^)	13.68	1.4	9	14	1.33	9	14.06	1.57	9	14	1.26	9
Agostini et al. (2017)	Jump (CMJ; cm)	35.8	6.97	15	34.27	6.08	15	50.93	7.47	15	44	7.64	15
	CODS (square agility test; s)	7.32	0.36	15	7.29	0.34	15	6.58	0.27	15	6.96	0.32	15
Alvarez et al. (2012)	Jump (CMJ; cm)	35.55	1.66	5	31.7	4.29	5	39.94	2.05	5	33.06	3.56	5
	Strength (1-RM Barbell squat; kg)	131.3	30.31	5	100.26	8.37	5	177.12	28.59	5	102.48	9.11	5
Behringer et al. (2013)	Strength (10RM Leg press; kg)	122.7	26	12	109.3	22.2	12	142.3	19.3	12	121.3	23.5	12
Berryman, Maurel & Bosquet (2010)	Jump (CMJ; cm)	33.3	4	11	34.2	3.6	11	35.3	3.6	11	35.2	4.2	5
	Endurance (3-km time trial; s)	748	81	11	711	107	5	712	76	11	690	109	5
Bishop et al. (2009)	Sprint (performance time to 5.5 m; s)	3.88	0.48	11	3.94	0.39	11	3.29	0.47	11	3.82	0.38	11
Blagrove et al. (2018)	Jump (CMJ; cm)	58.7	2.3	9	60.7	5.9	9	62.3	6.9	9	60.2	9.3	9
	Sprint (20-m sprint; s)	2.79	0.22	9	2.64	0.24	9	2.69	0.19	9	2.62	0.23	9
	Strength (MVC isometric squat; N.kg)	159.3	28	9	159.4	25.7	9	183.9	26.5	9	161.5	37.1	9
	Endurance (speed at VO2max; km.h)	16.8	2.4	9	17.8	0.8	9	17.3	2.6	9	17.8	1.7	9
Bogdanis et al. (2019)	Jump (CMJ; cm)	18.1	3.2	33	17	4.5	17	20.1	2.9	33	17.2	4.6	17
	Sprint (10-m sprint; s)	2.77	0.26	33	2.74	0.24	17	2.51	0.22	33	2.65	0.25	17
	CODS (5+5 m with 180° turn)	3.77	0.36	33	3.81	0.37	17	3.54	0.28	33	3.71	0.28	17
Chelly, Hermassi & Shephard (2015)	Jump (CMJ; cm)	23	3	14	21	3	13	25	3	14	22	3	13
	Sprint (Velocity at 5 m)	2	0.5	14	2.2	0.5	13	2.3	0.6	14	2.4	0.5	13
Cossor, Blanksby & Elliot (1999)	Sprint (50-m time swimming; s)	38.46	3.36	19	39.1	3.39	19	37.51	2.9	19	37.57	3.35	19
	CODS (5-m RTT; s)	7.45	0.72	19	7.63	0.64	19	6.94	0.67	19	6.89	0.65	19
Fernandez-Fernandez et al. (2016)	Jump (CMJ; cm)	30.1	4.3	24	30.3	4.3	27	32	4.1	24	30.9	4	27
	Sprint (5-m sprint; s)	1.17	0.1	24	1.16	0.1	27	1.11	0.1	24	1.15	0.1	27
	CODS (Agility 505 test; s)	2.95	0.2	24	2.93	0.1	27	2.86	0.2	24	2.92	0.1	27
Giovanelli et al. (2017)	Jump (CMJ; cm)	43.8	7.4	13	42.3	6.72	12	48.9	8.6	13	42.7	6.8	12
Hall, Bishop & Gee (2016)	Jump (CMJ; cm)	43.5	6.1	10	45.1	5.8	10	45.3	5.8	10	45.3	5.5	10
Li et al. (2019)	Jump (CMJ; cm)	31.06	3.41	10	33.46	4.27	9	34.51	3.85	10	34.26	4.22	9
	Sprint (50-m time; s)	6.25	0.19	10	5.94	0.21	9	6.11	0.24	10	5.92	0.3	9
	Strength (1 RM; kg)	60.25	8.03	10	63.33	9.35	9	70.5	11.17	10	64.44	8.82	9
	Endurance (5 km time; s)	953.7	12.3	10	954.11	6.75	9	926.9	9.92	10	947.33	10.03	9
Ludstrom, Betker & Ingraham (2017)	Jump (CMJ; cm)	51.3	12.7	11	57.6	19.6	11	50.3	11.4	11	55.4	14.2	11
	Sprint (200-m sprint run; s)	36.52	6.24	11	34.28	5.39	11	34.64	6.46	11	33.78	4.89	11
	Endurance (2 mile time trial; min)	14.7	2.4	11	14	2	11	14	2.3	11	13.3	1.7	11
Pellegrino, Ruby & Dumke (2016)	Jump (CMJ; cm)	44.7	4.1	11	48.8	4.2	11	44.4	4	11	45.5	4.6	11
	Endurance (3-km time trial; s)	780.9	29.9	11	830.4	35.6	11	760.8	29.1	11	817.2	39.8	11
Potdevin et al. (2011)	Jump (CMJ; cm)	28.92	4.82	12	27.04	4.51	11	32.45	4.2	12	25.88	3.82	11
	Sprint (25-m front crawl; m/s)	1.21	0.17	30	1.21	0.11	28	1.22	0.18	30	1.22	0.1	28
	Endurance (400-m front crawl; m/s)	0.92	0.1	12	0.88	0.08	11	0.96	0.09	12	0.89	0.06	11
Ramirez-Campillo et al. (2014)	Jump (CMJ; cm)	36.1	5.6	17	34.1	7.1	15	39.3	7	17	36.3	8.1	15
	Sprint (20-m; s)	3.92	0.3	17	3.97	0.2	15	3.83	0.3	17	3.94	0.4	15
	Endurance (2,4 time trial; min)	7.6	0.7	17	8	0.9	15	7.3	0.8	17	7.9	0.9	15
Redondo et al. (2014)	Jump (CMJ; cm)	36.02	1.84	6	32.15	3.4	6	38.58	2.28	6	31.43	4	6
	Strength (1RM squat)	88.6	30.74	6	80.27	25.63	6	111.22	34.75	6	81.12	25.91	6
Salonikidis & Zafeiridis (2008)	Jump (CMJ; cm)	13.6	4.4	16	14.9	2.6	16	18.3	6.1	16	14.7	2.8	16
	Sprint (12-m forward sprint; s)	2.47	0.2	16	2.47	0.19	16	2.44	0.22	16	2.41	0.19	16
	CODS (12-m sprint with turn; s)	2.7	0.17	16	2.73	0.14	16	2.68	0.19	16	2.68	0.14	16
	Strength (maximal isometric; N)	1,747	526	16	1,716	442	16	1,886	580	16	1,654	409	16
Saunders et al. (2006)	Jump (CMJ; cm)	41.2	9.8	7	39.9	6.9	8	44.6	5.8	7	40.1	3.1	8
	Strength (maximal dynamic; kg)	143.8	26.5	7	136.4	9.8	8	151.6	40.2	7	141.7	17.2	8
Sedano et al. (2013)	Jump (CMJ; cm)	30	3	6	32	2	6	33	3	6	33	2	6
	Strength (1RM squat; kg)	202.51	16.31	6	206.6	17.66	6	222	17.05	6	210.55	15.79	6
Turner, Owings & Schwane (2003)	Jump (CMJ; cm)	36	7	10	42	9	8	38	7	10	42	10	8
Vlachopoulos et al. (2018) (cyclists)	Jump (CMJ; cm)	42.7	5.9	14	45.4	7.8	12	45.9	6.5	14	46.1	8.2	12
	Endurance (20-m shuttle run; shuttles)	82.4	24.2	14	82.8	19.2	12	88.5	25.2	14	85.6	20.8	12
Vlachopoulos et al. (2018) (swimmers)	Jump (CMJ; cm)	46.8	7.2	19	46.5	9.4	18	49.9	7.6	19	45.9	9.7	18
	Endurance (20-m shuttle run; shuttles)	79.2	17.6	19	74.2	24.4	18	85.7	19.3	19	76.1	25.8	18
Wilson et al. (1993)	Jump (CMJ; cm)	35.8	6.7	16	37.2	8.2	16	39.5	9	16	38	8.2	16
	Sprint (30-m sprint; s)	4.61	0.4	16	4.73	0.58	16	4.6	0.38	16	4.77	0.58	16
	Strength (Maximal isometric force; N)	1,898	622	16	2,226	930	16	1,911	567	16	2,165	928	16

**Notes:**

α Before and after values denotes the mean ± standard deviation for each group before and after the intervention, respectively.

Abbreviations descriptions ordered alphabetically. 1RM, one repetition maximum; 10RM, 10 repetition maximum; CODS, change of direction speed; CMJ, countermovement jump; KE, knee extensors; MVC, maximal voluntary contraction; RTT, round trip time; VOmax, maximum volume of oxygen consumption; vVO2peak, velocity at peak volume of oxygen consumption.

### Methodological quality

Using the PEDro checklist, 4 studies achieved 4–5 points and were classified as being of “moderate” quality, while 22 studies achieved 6–10 points and were therefore considered as being of “high” methodological quality (Table 4).

**Table 4:** Physiotherapy evidence database (PEDro) scale ratings.

	N° 1*	N° 2	N° 3	N° 4	N° 5	N° 6	N° 7	N° 8	N° 9	N° 10	N° 11	Total (from a possible maximal of 10)
Ache-Dias et al. (2016)	1	1	0	1	0	0	0	1	1	0	1	6
Agostini et al. (2017)	1	1	0	1	0	0	0	1	1	1	1	6
Alvarez et al. (2012)	1	1	0	1	0	0	0	1	1	1	1	7
Behringer et al. (2013)	1	1	1	1	0	0	0	1	1	1	1	8
Berryman, Maurel & Bosquet (2010)	1	1	0	1	0	0	0	1	1	1	1	7
Bishop et al. (2009)	0	1	0	1	0	0	0	1	1	1	1	6
Blagrove et al. (2018)	1	1	1	1	0	0	0	1	1	1	1	8
Bogdanis et al. (2019)	1	1	0	1	0	0	0	1	1	1	1	7
Chelly, Hermassi & Shephard (2015)	1	1	0	1	0	0	0	1	1	1	1	6
Cossor, Blanksby & Elliot (1999)	0	0	0	1	0	0	0	1	1	1	1	5
Fernandez-Fernandez et al. (2016)	1	0	0	1	0	0	1	1	1	1	1	6
Giovanelli et al. (2017)	1	1	1	1	0	0	0	1	1	1	1	7
Hall, Bishop & Gee (2016)	1	1	0	1	0	0	0	1	1	1	1	6
Li et al. (2019)	1	0	0	1	0	0	0	1	1	1	1	5
Ludstrom, Betker & Ingraham (2017)	1	1	1	1	0	0	0	1	1	1	1	7
Pellegrino, Ruby & Dumke (2016)	1	1	0	1	0	0	0	1	1	1	1	6
Potdevin et al. (2011)	0	1	0	1	0	0	0	1	1	1	1	6
Ramirez-Campillo et al. (2014)	1	1	1	1	0	0	0	1	1	1	1	7
Redondo et al. (2014)	0	1	0	1	0	0	0	1	1	1	1	6
Salonikidis & Zafeiridis (2008)	0	1	0	1	0	0	0	1	0	1	1	5
Saunders et al. (2006)	0	1	0	1	0	0	0	1	1	1	1	6
Sedano et al. (2013)	1	1	0	1	0	0	0	1	1	1	1	6
Turner, Owings & Schwane (2003)	0	1	0	1	0	0	0	1	1	1	1	6
Vlachopoulos et al. (2018)	1	1	1	1	0	0	0	1	1	1	1	7
Wilson et al. (1993)	1	1	0	0	0	0	0	1	1	0	1	4

**Note:**

* A detailed explanation for each PEDro scale item can be accessed at https://www.pedro.org.au/english/downloads/pedro-scale (access for this review: 27 May 2020).

### Meta-analysis results

#### Jump performance

**Figure 2:**
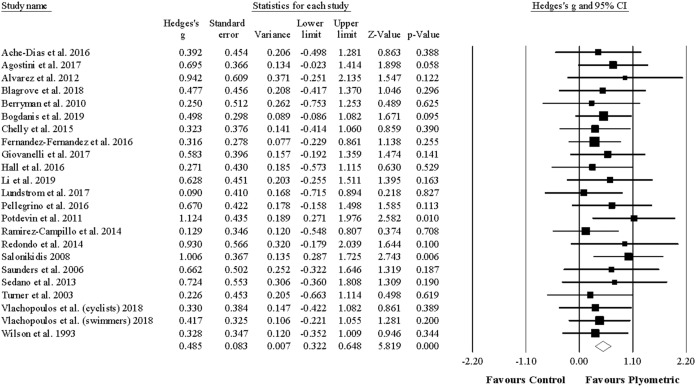
Forest plot of changes in vertical jump performance. Forest plot of changes in vertical jump performance, in athletes participating in plyometric jump training compared to controls. Values shown are effect sizes (Hedges’s g) with 95% confidence intervals (CI). The size of the plotted squares reflects the statistical weight of the study.

Twenty-two studies provided data for jump (vertical) performance, involving 23 experimental and 23 control groups (pooled *n* = 567). There was a significant effect of PJT on vertical jump performance (ES = 0.49; 95% CI [0.32–0.65]; *p* < 0.001; *I*^2^ = 0.0%; Egger’s test *p* = 0.117; Fig. 2). The relative weight of each study in the analysis ranged from 1.9% to 9.0%.

#### Linear sprint performance

**Figure 3:**
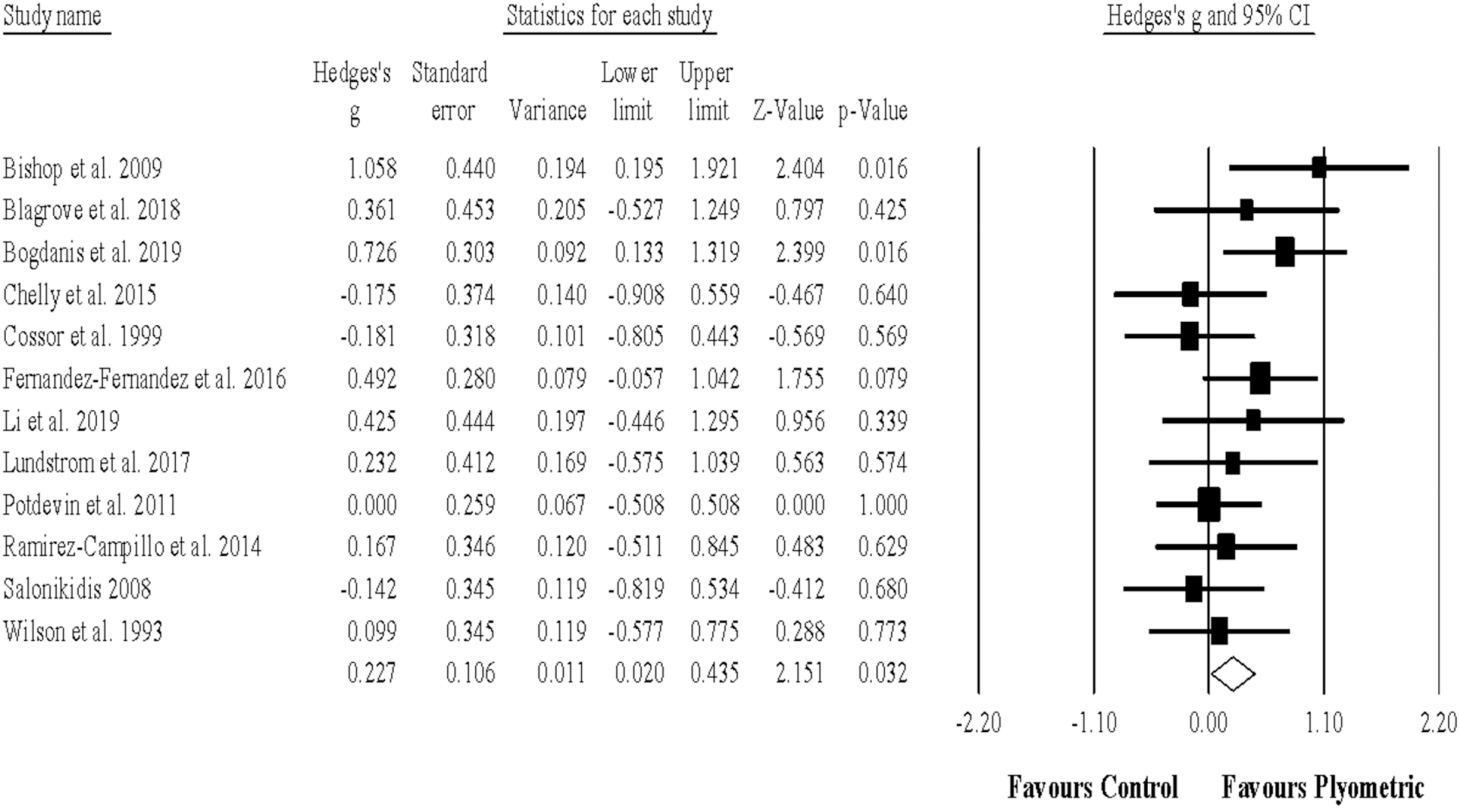
Forest plot of changes in linear sprint performance. Forest plot of changes in linear sprint performance, in athletes participating in plyometric jump training compared to controls. Values shown are effect sizes (Hedges’s g) with 95% confidence intervals (CI). The size of the plotted squares reflects the statistical weight of the study.

Twelve studies provided data for sprint (linear) performance, involving 12 experimental and 12 control groups (pooled *n* = 401). There was a significant effect of PJT on sprint performance (ES = 0.23; 95% CI [0.02–0.44]; *p* = 0.032; *I*^2^ = 10.9%; Egger’s test *p* = 0.518; Fig. 3). The relative weight of each study in the analysis ranged from 5.1% to 13.7%.

#### Sprint with change of direction performance

**Figure 4:**
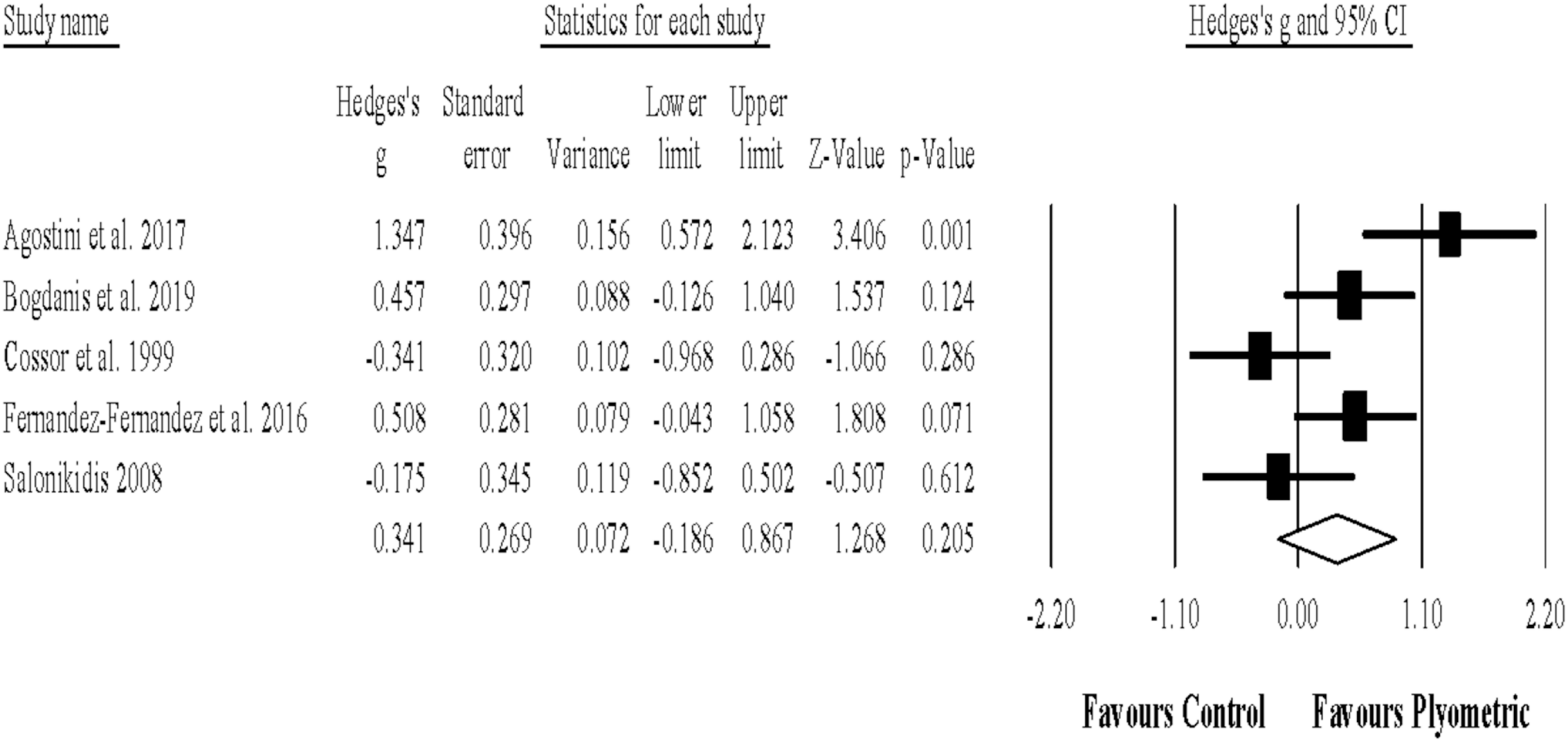
Forest plot of changes in sprint with change of direction performance. Forest plot of changes in sprint with change of direction performance, in athletes participating in plyometric jump training compared to controls. Values shown are effect sizes (Hedges’s g) with 95% confidence intervals (CI). The size of the plotted squares reflects the statistical weight of the study.

Five studies provided data for sprint with COD performance, involving 5 experimental and 5 control groups (pooled *n* = 201). There was a non-significant effect of PJT on sprint with COD performance (ES = 0.34; 95% CI [−0.19 to 0.87]; *p* = 0.205; *I*^2^ = 70.9%; Egger’s test *p* = 0.657; Fig. 4). The relative weight of each study in the analysis ranged from 17.6% to 21.7%.

#### Strength

**Figure 5:**
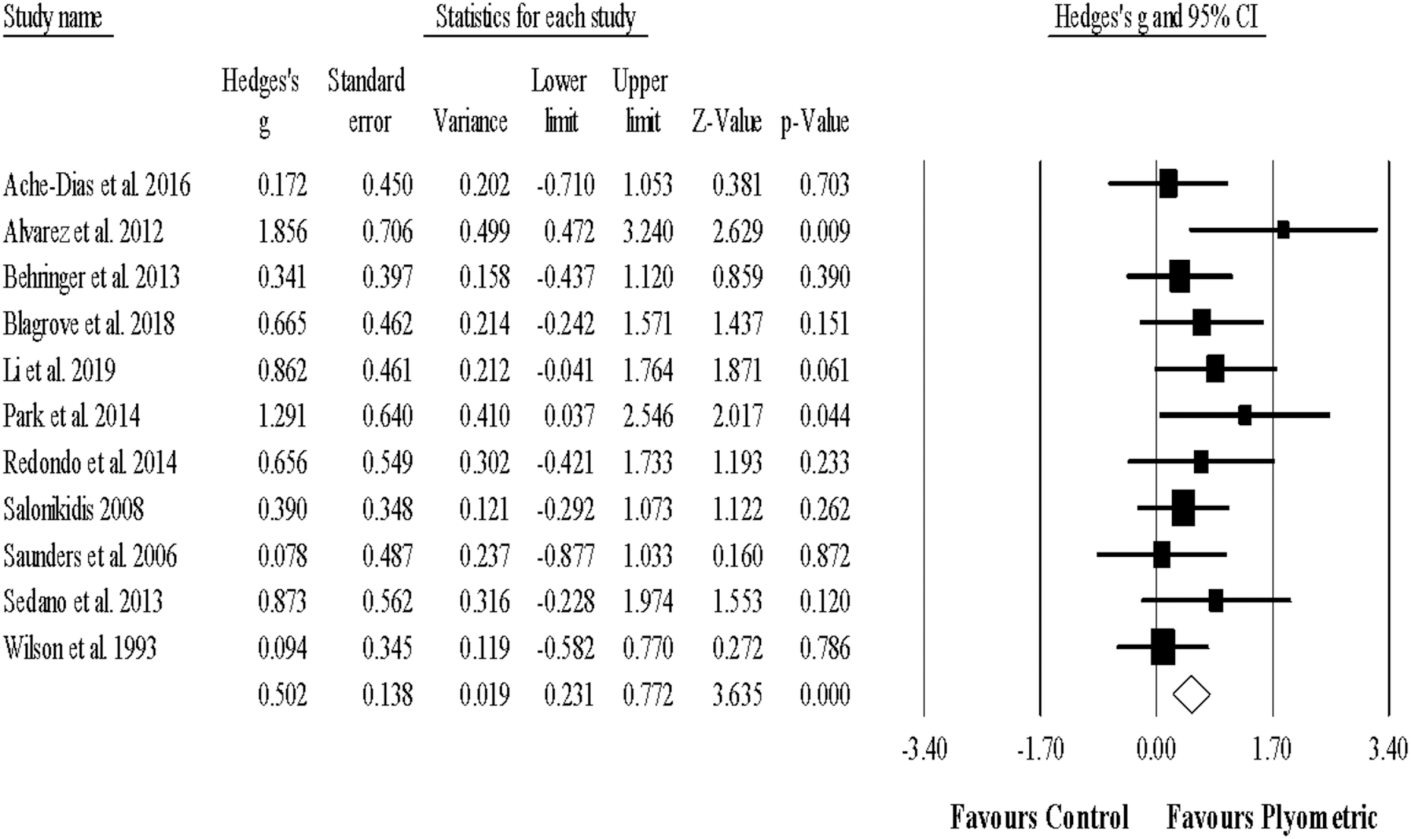
Forest plot of changes in strength (maximal) performance. Forest plot of changes in strength (maximal) performance, in athletes participating in plyometric jump training compared to controls. Values shown are effect sizes (Hedges’s g) with 95% confidence intervals (CI). The size of the plotted squares reflects the statistical weight of the study.

Eleven studies provided data for strength (maximal) performance, involving 11 experimental and 11 control groups (pooled *n* = 202). There was a significant effect of PJT on strength performance (ES = 0.50; 95% CI [0.23–0.77]; *p* < 0.001; *I*^*2*^ = 0.0%; Egger’s test *p* = 0.004 Fig. 5). After adjusting according to the Duval and Tweedie´s method ES = 0.38 (95% CI [0.08–0.68]). The relative weight of each study in the analysis ranged from 3.8% to 16.0%.

#### Endurance

**Figure 6:**
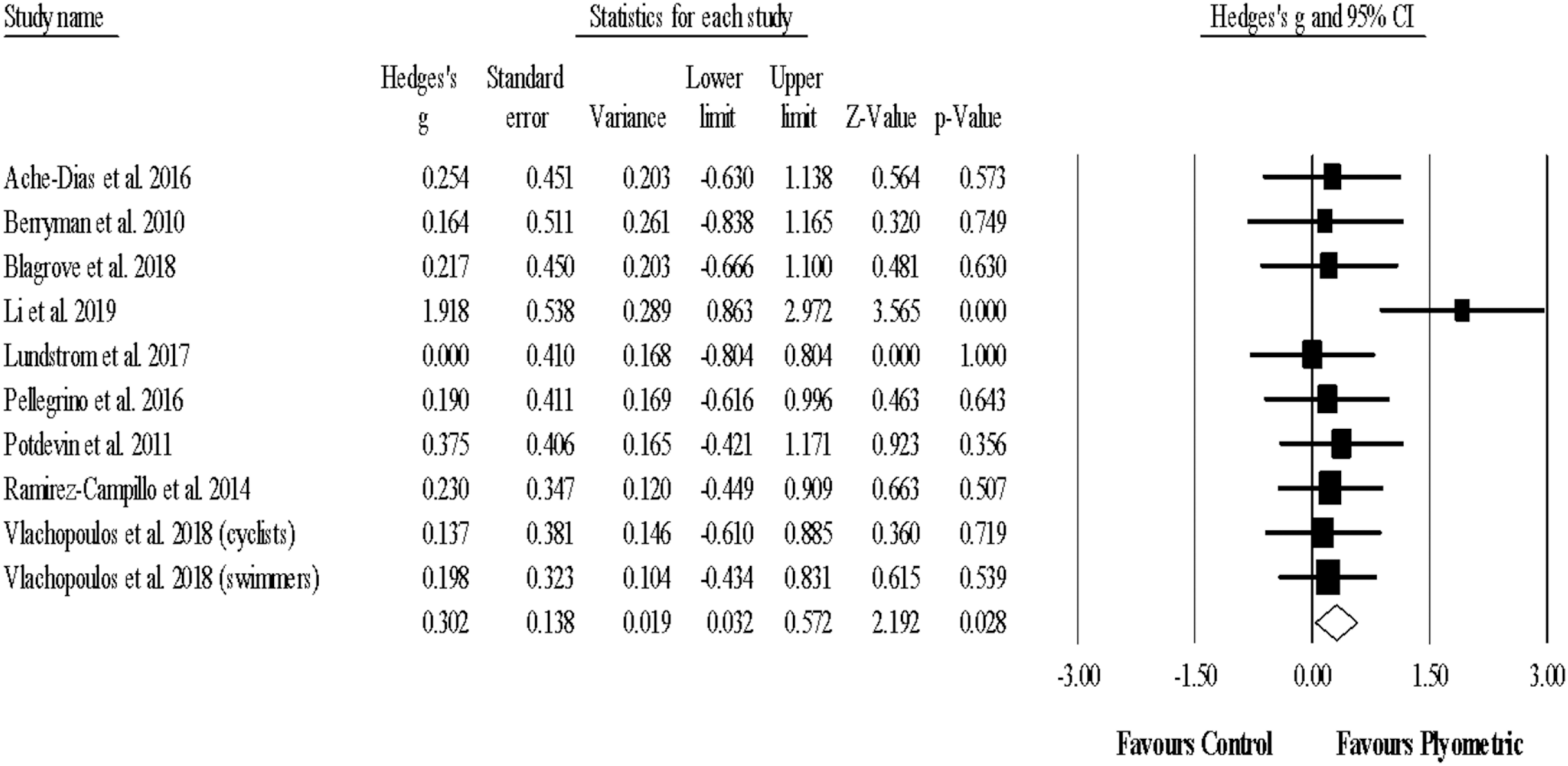
Forest plot of changes in endurance performance. Forest plot of changes in endurance performance, in athletes participating in plyometric jump training compared to controls. Values shown are effect sizes (Hedges’s g) with 95% confidence intervals (CI). The size of the plotted squares reflects the statistical weight of the study.

Nine studies provided data for endurance performance, involving 10 experimental and 10 control groups (pooled *n* = 233). There was a significant effect of PJT on strength performance (ES = 0.30; 95% CI [0.03–0.57]; *p* = 0.028; *I*^2^ = 11.1%; Egger’s test *p* = 0.119, Fig. 6). The relative weight of each study in the analysis ranged from 6.1% to 15.2%.

### Subgroup analyses

Regarding vertical jump performance, similar improvements were noted for male (ES = 0.48; 11 studies), female (ES = 0.51; three studies) and mixed male-female (ES = 0.42; 7 studies) athletes (*p* = 0.931). In addition, similar improvements were noted for youth (ES = 0.47; nine studies) and adult (ES = 0.50; 14 studies) athletes (*p* = 0.857). Further, similar improvements (*p* = 0.657) were noted for gymnasts (ES = 0.51; three studies), runners (ES = 0.40; 12 studies), and athletes from other (i.e., golf; tennis; swimming; fencing; cycling; resistance training) sports (ES = 0.57; eight studies).

Regarding linear sprint performance, similar improvements were noted for male (ES = 0.28; three studies) and mixed male-female (ES = 0.06; five studies) athletes (*p* = 0.394). Of note, only one study reported data for female linear sprint performance (not included in the analyses). In addition, similar improvements were noted for youth (ES = 0.30; seven studies) and adult (ES = 0.13; five studies) athletes (*p* = 0.473). Further, similar improvements (*p* = 0.837) were noted for swimmers (ES = 0.22; three studies), runners (ES = 0.17; five studies) and athletes from other (i.e., tennis; gymnasts; resistance training) sports (ES = 0.33; four studies).

Regarding maximal strength performance, a reduced number of studies available precluded a subgroup analysis according to the sex and age categories. Similar improvements (*p* = 0.873) were noted for runners (ES = 0.51; five studies) and athletes from other (i.e., golfers; fencers; tennis; resistance training) sports (ES = 0.46; five studies).

Regarding endurance performance, similar improvements were noted for male (ES = 0.54; four studies) and mixed male-female (ES = 0.21; six studies) athletes (*p* = 0.422). Of note, no study reported data for female endurance performance. In relation with athletes age category, similar improvements were noted for youth (ES = 0.23; four studies) and adult (ES = 0.40; six studies) athletes (*p* = 0.578). Further, similar improvements (*p* = 0.641) were noted for runners (ES = 0.37; seven studies) and athletes from other (i.e., swimming; cycling) sports (ES = 0.23; three studies).

Regarding type of test, similar improvements were noted for short-duration sprint tests (ES = 0.33; nine studies) and longer-duration tests (ES = −0.01; three studies) (*p* = 0.124). In addition, similar improvements were noted for isometric maximal strength test (ES = 0.33; three studies) and dynamic tests (ES = 0.56; seven studies) (*p* = 0.427).

### Adverse effects

Among the included studies, none reported soreness, pain, fatigue, injury, damage or adverse effects related to the PJT intervention.

## Discussion

### Summary of main results

Improvements in vertical jump, linear sprint, strength and endurance performance were observed in ISA after PJT compared to a control condition. However, no significant effect was found for COD performance. These conclusions are based on studies that were generally classified as being of high methodological quality.

### Jump performance

The main findings of this study indicate enhancements on proxies of muscle power (CMJ) among ISA, concurring with the wider strength and conditioning literature available for other sports, mainly team sports (Ramirez-Campillo et al., 2020a, 2020d; Van de Hoef et al., 2019). A recent meta-analysis (Ramirez-Campillo et al., 2020b) revealed that PJT improved CMJ height in volleyball players, regardless of players’ age and sex. Considering that volleyball players are routinely involved in jumping actions, is not surprising that ISA, such as swimmers, with a relatively low training age regarding jumping actions (thus greater ceiling for improvement) achieved a significant improvement in such actions after PJT. However, the improvement observed in the current SRMA was small (ES = 0.49). Independent of this, jumping performance improvement can generally be attributed to factors such as enhanced motor unit recruitment, greater inter-muscular coordination, enhanced neural drive to agonist muscles, better utilization of the SSC (Markovic & Mikulic, 2010; Taube, Leukel & Gollhofer, 2012), and probably selective muscle hypertrophy (Grgic, Schoenfeld & Mikulic, 2020), factor that may relate to ISA performance.

### Linear and change of direction speed

We found small improvements in linear sprint (ES = 0.23) in response to PJT. Neuro-mechanical adaptations induced by PJT (e.g., enhanced neural drive to agonist muscles, alterations to muscle-tendon stiffness) (De Villarreal, Requena & Cronin, 2012; Haugen et al., 2019; Markovic & Mikulic, 2010) may improve SSC efficacy. Because of improvements in SSC efficacy, a greater force would be produced in the concentric phase of the movement after a rapid eccentric muscle action (Komi & Gollhofer, 1997; Markovic & Mikulic, 2010; Radnor et al., 2017), a key requirement for better sprint performance (Bishop & Girard, 2013). Furthermore, improved neuro-mechanical properties after PJT may enhance different ground reaction force (GRF) characteristics (e.g., impulse; peak force) (Markovic & Mikulic, 2010), in turn contributing to faster sprint acceleration (Lockie et al., 2014). In contrast to linear sprinting speed performance, current findings indicate that PJT interventions that lasted between 8 up to 48 weeks, with 2–3 sessions per week, induced no significant improvement in COD compared to controls. However, a small improvement (ES = 0.34) in COD in favor of the PJT groups over the controls was indeed observed, with a 95%CI that ranged between ES = −0.19 up to ES = 0.87. Considering the relevance of both vertical and horizontal force neuromuscular-generating capabilities (Young & Farrow, 2006; Young, 2006; Young, Dawson & Henry, 2015), and the relevance of unilateral performance during COD movements (Ramirez-Campillo et al., 2015; Young, James & Montgomery, 2002), the incorporation of vertical, horizontal, and unilateral drills may increase the chances for COD improvements. Overall, it seems that small COD improvements can be expected after PJT interventions in ISA. However, if such improvement are of relevance for some ISA (e.g., endurance runners; cyclists; swimmers) is a future line of research inquiry.

### Strength

Maximal strength improved after PJT, with a small magnitude (ES = 0.30). These findings support data from previous studies examining the benefits of PJT for maximal strength (De Villarreal, Requena & Newton, 2010). Improvements in strength after PJT may be related to neural adaptations, including improved motor-unit firing frequency, synchronisation, excitability and efferent motor drive (Markovic & Mikulic, 2010). This can result in the optimization of the relative force generated per each motor unit recruited (Radnor et al., 2017). However, improvements in muscle strength after PJT may also be related to muscle hypertrophy (Grgic, Schoenfeld & Mikulic, 2020). The relative contribution of the aforementioned neuro-muscular factors may vary depending on the duration of the PJT interventions, and future studies may examine such relative contributions among ISA.

### Endurance

Our results demonstrated a significant (ES = 0.30) increase in endurance performance after PJT compared to controls. PJT may not induce a significant increase in underlying aerobic qualities such as maximal oxygen consumption (VO2max) (Barnes & Kilding, 2015; Blagrove, Howatson & Hayes, 2017) or lactate threshold (Blagrove, Howatson & Hayes, 2017; Gorostiaga et al., 2004), but has been shown to improve anaerobic performance qualities (Assuncao et al., 2017) related to endurance performance. Of note, this seems to be the first meta-analysis to report the positive effects of PJT on endurance performance among ISA. Improvements in explosive performance after PJT can contribute to running economy (Blagrove, Howatson & Hayes, 2017), independently from the influence on VO2max (Balsalobre-Fernandez, Santos-Concejero & Grivas, 2016) or lactate metabolism (Spurrs, Murphy & Watsford, 2003). Indeed, the improvement in jumping performance observed in this meta-analysis may reflect an improved performance to produce maximal strength in a minimal time (Flanagan & Comyns, 2008) as a consequence of improved rate-of-force development and motor unit recruitment level (Markovic & Mikulic, 2010). In turn, this may have transferred into improved running economy and enhance aerobic performance independently of others aerobic indicators (Coyle, 1995), especially considering the relevance of neuromuscular-mediated changes in the athletes’ running economy (Yamamoto et al., 2008). An increased tendon stiffness after PJT (Markovic & Mikulic, 2010), may also allow for a faster transfer of force from contracting muscles to moving bones through tendons (Legerlotz et al., 2016), positively affecting athletes’ running economy (Balsalobre-Fernandez, Santos-Concejero & Grivas, 2016; Barnes & Kilding, 2015). Direct assessment of potential mechanisms that could improve endurance performance after PJT in ISA deserves further consideration by well-controlled studies.

### Subgroup analyses

Our analyses indicated that sex, age and sport background had no modulator role on the effect of PJT in the vertical jump, linear sprint, maximal strength and endurance performance of ISA. In contrast to our findings, previous studies reported that the adaptive responses to PJT may be affected by participant age (Asadi et al., 2017; Moran et al., 2019; Moran et al., 2017), sex (De Villarreal et al., 2009) and sport background (Asadi et al., 2016; De Villarreal, Requena & Cronin, 2012; Fleck & Kraemer, 2004; Izquierdo et al., 2002; Taylor et al., 2018; Vlachopoulos et al., 2018). Indeed, one of the included studies in our meta-analysis (Vlachopoulos et al., 2018) reported that ISA (i.e., swimmers; cyclists) had significant improvements in vertical jump and endurance performance, while soccer players showed no improvement. Therefore, the reduced number of studies available for some moderators in our meta-analysis may partially explain the lack of significant effect for ISA sex, age and sport background on physical fitness adaptations after PJT. Further studies would be needed to identify optimal PJT strategies according to ISA sex, age and sport background.

Regarding type of test, similar improvements were noted for short-duration (i.e., 2–7 s) and longer-duration (i.e., 21–39 s) sprint tests. In addition, similar improvements were noted for dynamic and isometric maximal strength tests. Such findings are similar to those previously reported for a mixed sample of ISA and team sport athletes (De Villarreal, Requena & Cronin, 2012; De Villarreal, Requena & Newton, 2010). Indeed, improvements of ES = 0.20-0.39 were noted for sprint distances from 10-m up to 100-m, and for maximal strength tests involving isometric and dynamic actions (De Villarreal, Requena & Cronin, 2012; De Villarreal, Requena & Newton, 2010). However, in our meta-analysis, the small improvement for shorter sprinting distances (ES = 0.33) contrast with the trivial detrimental effect (ES = −0.01) for longer distances. Moreover, different maximal strength testing protocols have reported different results (Gentil et al., 2017). Considering the short-duration and dynamic nature of PJT drills, a greater transfer of adaptations derived from PJT would be specked for short duration sprint distances and dynamic maximal strength actions (Loturco et al., 2015, 2014; Ramirez-Campillo et al., 2019). Although our findings do not support the prescription of a specific testing protocol, according to the ISA needs, specific protocols might be selected to better reflect the competitive demands of the athlete and the potential transference effects from PJT.

### Methodological quality of included studies

Although all included studies in our meta-analysis were classified as being of moderate-high quality, most scored no more than six points in the PEDro scale. Previous systematic reviews that focused on PJT (Bedoya, Miltenberger & Lopez, 2015; Johnson, Salzberg & Stevenson, 2011; Stojanović et al., 2017) and used the PEDro scale also suggested that published studies in this area are generally of medium quality. This is probably due to the difficulties in conducting studies that include blinding of participants or therapists. Relatedly, a recent PJT scoping review (Ramirez-Campillo et al., 2020c) highlighted several methodological shortcomings from 420 analysed studies, in particular an incomplete description of training intervention characteristics. Even though the included studies in the current SRMA generally provided a clear description of the training intervention, some key elements, such as the recovery time between sets and repetitions (or even between training sessions), was not clearly reported in most studies. Future studies should strive for a more robust methodological approach.

### Limitations

Firstly, the limited number of studies providing moderator data according to ISA sex, age and sport background for the outcomes vertical jump, linear and change of direction speed, maximal strength and endurance, precluded firm conclusions regarding the potential role of these moderators on the physical fitness adaptations to PJT. Secondly, the included studies did not reported adverse effects (e.g., injuries, pain). However, due to the lack of reported information, these statements must be viewed with caution. Therefore, a cautious approach is recommended, initially including moderate PJT loads and adequate progression, particularly for those unexperienced with PJT and/or with an insufficient strength and conditioning base.

In conclusion, this meta-analysis found that PJT induces small improvements on ISA physical fitness, including jumping, sprinting speed, strength and endurance. Such findings were found among ISA such as runners, weightlifters, gymnasts, golfers, swimmers, throwers, and fencers. Moreover, the PJT programmes lasted a median of 8.5 weeks. For most studies, the neuromechanically mechanisms underlying the physical fitness improvements were not addressed. Therefore, further studies are needed to clarify the optimal dose of PJT according to the particular sport practiced by ISA. In addition, more long-term studies (i.e., >12 weeks) are needed in order to clarify the effects of PJT embedded in comprehensive multidimensional long-term training programs for ISA. Finally, future studies must sought to clarify the underlying mechanisms responsible for the physical fitness improvements noticed after PJT in ISA.

## Supplemental Information

10.7717/peerj.11004/supp-1 Supplemental Information 1 Contributions to the advancement of the field.

10.7717/peerj.11004/supp-2 Supplemental Information 2 PRISMA checklist.

## Additional Information and Declarations

### Data Availability

The following information was supplied regarding data availability: Raw measurements are available in Table 3.
