# Supplementary material for: Plyometric jump training effects on the physical fitness of individual-sport athletes: a systematic review with meta-analysis
Source: PeerJ. 2021 Mar 1;9:e11004. doi: 10.7717/peerj.11004 (PMC7931718; doi:10.7717/peerj.11004)
Supplement: Supplemental Information 1 [file peerj-09-11004-s001.docx]

The rationale for conducting the systematic review / meta-analysis.

Physical fitness is of critical importance to several sports, particularly in individual sports. Plyometric jump training (PJT), through enhancement of the stretch-shortening cycle and associated neuro-mechanical mechanisms, may facilitate improvements in physical fitness. There is compelling evidence showing that PJT may improve physical fitness regardless of age and sex. However, the studies that explored the effects of PJT on the physical fitness in individual sport athletes (ISA) have reported inconsistent results, with benefits in some, but not all studies. Therefore, we aimed to conduct a systematic review with meta-analysis to explore (and clarify) the effects of PJT on the physical fitness of ISA.

The contribution that it makes to knowledge in light of previously published related reports, including other meta-analyses and systematic reviews

Most PJT intervention studies includes relatively small sample size. A 2020 PJT scoping review including 420 articles reported that an average of ~10 participants was included per study. In PJT studies conducted among ISA, the average sample size was ~13 participants per study, suggesting that some studies may be statistically underpowered to find significant effects. Moreover, even if significant effects emerge from studies with an small sample size, their replicability would be limited. This problem of underpowered studies may partially be resolved by conducting a meta-analysis. To date, several systematic reviews and meta-analysis that assess the effects of PJT on different components of physical fitness have been published. These analyses have provided evidence that PJT is effective for inducing large improvements in vertical jump ability, strength, and sprint capacity or without change of direction. To date, however, no previous meta-analysis has systematically explored the effects of PJT on the physical fitness of ISA. Moreover, previous meta-analyses incorporated participants across a range of different sports. Because the effects of PJT may vary depending on the sports background of the athlete, findings from these studies cannot be generalised to ISA.

The results derived from our systematic review and meta-analysis may help practitioners to sustain more robust training interventions for the improvement of ISA physical fitness.
